# Influence of Silica Specific Surface Area on the Viscoelastic and Fatigue Behaviors of Silica-Filled SBR Composites

**DOI:** 10.3390/polym13183094

**Published:** 2021-09-14

**Authors:** Hiron Raja Padmanathan, Carlos Eloy Federico, Frédéric Addiego, Robert Rommel, Ondřej Kotecký, Stephan Westermann, Yves Fleming

**Affiliations:** 1 Department of Materials Research and Technology, Luxembourg Institute of Science and Technology, 5 Avenue des Hauts-Fourneaux, L-4362 Esch-sur-Alzette, Luxembourg; hironraja.padmanathan@list.lu (H.R.P.); carloseloy.federico@list.lu (C.E.F.); frederic.addiego@list.lu (F.A.); stephan.westermann@list.lu (S.W.); 2 Department of Physics and Materials Science, University of Luxembourg, 2 Avenue de l’Université, L-4365 Esch-sur-Alzette, Luxembourg; 3 Goodyear Innovation Center Luxembourg (GIC*L), Avenue Gordon Smith, L-7750 Colmar-Berg, Luxembourg; robert_rommel@goodyear.com (R.R.); ondrej_kotecky@goodyear.com (O.K.)

**Keywords:** elastomer, fatigue, viscoelasticity, crack initiation, silica, specific surface area

## Abstract

This work aimed at studying the effect of a silica specific surface area (SSA), as determined by the nitrogen adsorption method, on the viscoelastic and fatigue behaviors of silica-filled styrene–butadiene rubber (SBR) composites. In particular, silica fillers with an SSA of 125 m^2^/g, 165 m^2^/g, and 200 m^2^/g were selected. Micro-computed X-ray tomography (µCT) was utilized to analyze the 3D morphology of the fillers within an SBR matrix prior to mechanical testing. It was found with this technique that the volume density of the agglomerates drastically decreased with decreasing silica SSA, indicating an increase in the silica dispersion state. The viscoelastic behavior was evaluated by dynamic mechanical analysis (DMA) and hysteresis loss experiments. The fatigue behavior was studied by cyclic tensile loading until rupture enabled the generation of Wöhler curves. Digital image correlation (DIC) was used to evaluate the volume strain upon deformation, whereas µCT was used to evaluate the volume fraction of the fatigue-induced cracks. Last, scanning electron microscopy (SEM) was used to characterize, in detail, crack mechanisms. The main results indicate that fatigue life increased with decreasing silica SSA, which was also accompanied by a decrease in hysteresis loss and storage modulus. SEM investigations showed that filler–matrix debonding and filler fracture were the mechanisms at the origin of crack initiation. Both the volume fraction of the cracks obtained by µCT and the volume strain acquired from the DIC increased with increasing SSA of silica. The results are discussed based on the prominent role of the filler network on the viscoelastic and fatigue damage behaviors of SBR composites.

## 1. Introduction

Over the last decades, much progress has been made in understanding the mechanisms of the fatigue of filled rubber compounds [1,2]. The addition of fillers such as carbon black (CB) and silica, also known as reinforcements, improve the mechanical properties [3,4,5]. At the same time, the addition of these fillers introduces a non-linear dependency upon the dynamic strain amplitude [6,7]. This includes stress softening due to the Mullins effect, Payne effect, and hysteresis. Hysteresis influences self-heating upon deformation and, thus, the product’s mechanical performance [8]. The lifetime of a usual rubber product under dynamic loading during operation consists of a crack initiation stage [9] followed by a crack growth stage, which ultimately leads to the integrity loss of the product [10,11]. In styrene–butadiene rubber (SBR), the identification of the mechanisms responsible for the fatigue behavior requires knowledge about the composition of the butadiene microstructure [10]. Understanding the mechanism of crack initiation and fatigue behavior is necessary for enhancing compound durability to produce further improved products.

One approach to understanding fatigue behavior relies on performing fatigue testing until fracture [12]. In this context, the end of product life can be defined as the number of cycles needed to attain a certain level of stiffness loss [13] or the initiation of a crack of a given size [12,13]. Many factors influence the material’s fatigue behavior, including compound formulation, load history, and environmental factors, as well as the dissipative behavior of the material [14]. The role of the latter is critical for understanding and improving fatigue properties. The dissipative behavior includes softening due to the Mullin’s effect [15,16] and strain-induced crystallization [17], as well as the material’s viscoelasticity and hysteresis. It is known that elastomers have viscoelastic characteristics resulting in the loss of energy during loading and unloading. The compound’s energy loss contributes to hysteresis and the compound’s temperature rise. Hysteresis is an irreversible process during which mechanical energy is transformed into heat [18,19].

Currently, precipitated silica is widely used as a reinforcing filler in tires [20]. However, silica on its own is not effective as a reinforcing agent due to its limited chemical interaction with rubber caused by the silica surface’s hydrophilic silanol groups. Therefore, silica is used, along with a silane coupling agent, to reduce the filler–filler contact [21], as well as to prevent the adsorption of curatives on the silica surface [22] and, consequently, to improve the material’s mechanical properties. Dynamic mechanical analysis (DMA) has largely been used to study the material’s viscoelastic properties with respect to strain, temperature, and frequency [23]. In the case of filled elastomers, the filler–filler network is responsible for the material’s non-linear viscoelastic behavior [8]. In a number of studies, the macroscopic strain field of the samples was characterized using the digital image correlation technique (DIC) [24,25], which helped access the volume change upon deformation [26,27]. In these works, the volume change was correlated to the crack growth and the fatigue properties.

Based on microscopic approaches, some studies showed that rubber compounds contain heterogeneities that were responsible for crack initiation [28] and led to loss of the compound’s stiffness. Upon loading, crack precursors cause local stress fields and tend to facilitate crack growth [29]. The heterogeneities responsible for the detriment of properties were considered as crack precursors [28,30]. Hao Guo et al. [31] investigated the crack precursors by means of tensile and tear tests, showing that a comparative study of crack precursors can be performed using scanning electron microscopy (SEM).

In many studies, the fatigue behavior of these compounds has been investigated by means of structural characterization techniques, including SEM [32], micro-computed X-ray tomography (µCT) [33,34,35], and small-angle X-ray scattering [36]. Huneau et al. [2] investigated the fatigue crack initiation on CB-filled natural rubber and revealed that the predominant crack initiation sites were around CB agglomerates. The authors concluded that debonding occurred at the rubber–CB interface, mainly around the CB agglomerate’s poles in the stretching direction, as the main mechanism of initiation. This is mainly due to the limited interfacial adhesion between the CB agglomerates and the rubber matrix. Crack initiation due to ZnO particles was also reported in the same work. They observed two mechanisms of crack initiation: one due to the matrix–ZnO debonding (interfacial separation) and one due to the fracture of a ZnO inclusion.

An hourglass-shaped sample geometry was widely used to understand fatigue crack initiation. This is mainly for the strain gradient induced by the geometry, promoting crack initiation under the surface of the sample’s median section [37]. Such surface cracks can be investigated using SEM, a technique that provides a high spatial resolution and chemical contrast. However, a critical drawback of using SEM is that only surface information can be retrieved, whereas several cracks are likely to be initiated in the bulk.

µCT has become a promising technique in investigating crack initiation and growth in the bulk of filled rubber compounds subjected to fatigue loading. Marco et al. [38] used µCT to study the initiation and growth of cavities on polychloroprene rubber. In their work, information about the percentage of cycle required for the observation of early cavity initiation and damage parameters like porosity and defect volume density was proposed.

Our previous studies on fatigue crack initiation in silica-filled SBR revealed that the cracks were initiated in the bulk and on the sample surface [24]. This finding required the use of techniques capable of analyzing crack initiation in these two regions, as the combination of SEM for surface analysis and µCT for bulk analysis. In addition, to our best knowledge, fatigue crack initiation mechanisms and fatigue life in silica-filled elastomers have never been investigated as a function of silica specific surface area (SSA). Therefore, in this work, the influence of silica SSA on the viscoelastic and fatigue properties of silica-filled styrene–butadiene rubber (SBR) composites was examined. Concerning fatigue testing, Wöhler curves were generated, whereas fatigue-induced damage mechanisms were observed using SEM and µCT. On the other hand, DMA and hysteresis loss experiments were performed to understand the compound’s viscoelastic properties. This work’s main finding showed that an increase in silica SSA decreases the fatigue end life due to a decrease in the silica dispersions state, which also drastically influences viscoelastic properties.

## 2. Experimental Section

### 2.1. Materials, Mixing and Curing

**Figure 1:**
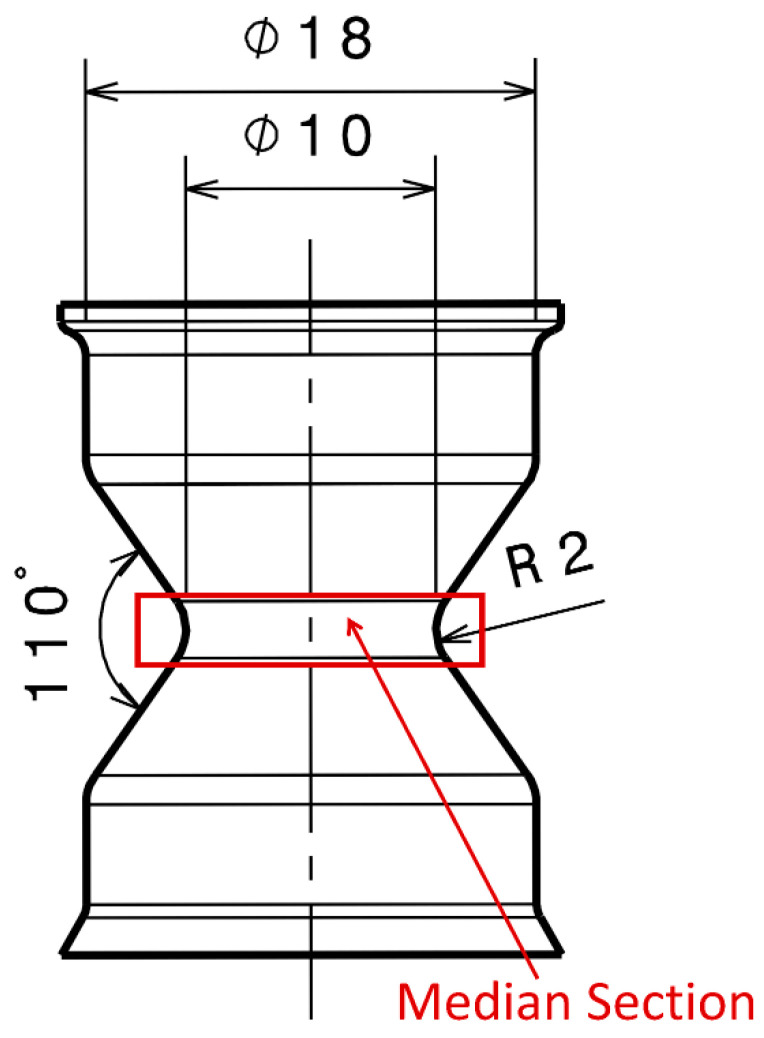
Hourglass sample with its median section exhibiting the highest surface strain (highlighted with the red rectangle) upon loading.

The compound formulations in units of parts per hundred parts of rubber (phr) are presented in Table 1. The amounts of silane Si266 (Evonik Corp., Parsippany, NJ, USA) and accelerators were adjusted for the three compounds based on the silica’s SSA to have a similar SBR/silica contact area and adsorption behavior on the silica surface [39,40]. Note that the Brunauer–Emmett–Teller (BET) method was used with nitrogen (N_2_) as a gaseous adsorbate to determine the silica SSA. For this study, rubber sheets of dimensions 100 mm × 100 mm × 2 mm, as well as hourglass samples (geometry depicted in Figure 1), were prepared by compression molding and transfer molding, respectively. The samples were cured at 170 °C for 10 min.

The hourglass-shaped samples (Figure 1) were used to perform fatigue testing. The advantage of choosing this sample geometry is that the crack initiation zone is well localized. Indeed, when deformed, the sample’s median section experiences large surface strains in comparison to the strains experienced in the bulk region. For the sake of simplicity, we refer to the compounds with a silica SSA of 125 m^2^/g, 165 m^2^/g, and 200 m^2^/g as C1, C2, and C3, respectively.

### 2.2. Tensile Testing

The uniaxial tensile testing was carried out using dumbbell-shaped samples (ISO-37-2 type-2) cut out from rubber sheets with a thickness of 2 mm. These samples were tested with an electro-mechanical machine (Instron 5967, Norwood, MA, USA) with a load cell capacity of 1 kN. All the tests were performed at 21 ± 2 °C and at a grip separation rate of 200 mm/min. Stress and strain were measured continuously up to integrity loss. Each sample’s strain energy density was calculated by integrating the stress–strain curve.

### 2.3. Critical Tearing Energy

For each compound, tear testing was performed using a nicked angle tear specimen [31]. The measurement was carried out with the Instron 5967 machine at a testing rate of 500 mm/min. The tear strength was calculated based on the following equation:

(1) $$T_{s}=\frac{F}{d}$$

where $T_{s}$ is the tear strength (N/mm), *F* is the maximum force at rupture (N), and *d* is the thickness of the sample (mm).

The crack precursor size of the samples can be calculated based on the equation [31]:

(2) $$C_{0}=\frac{T_{s}\;\sqrt{1+\varepsilon_{b}}}{\pi \sigma_{b}\varepsilon_{b}}$$

where $C_{0}$ is the crack precursors size (µm) and $\sigma_{b}$ and $\varepsilon_{b}$ are the stress (MPa) and strain (%) at break obtained from the quasi-static testing, respectively.

### 2.4. Hysteresis and Volume Strain

To compare the hysteresis loss of the different compounds, the hourglass specimen samples were subjected to cyclic loading with the Instron 5967 machine at a crosshead speed of 200 mm/min. The tests were displacement-controlled, and the samples were subjected to 100%, 130%, and 150% of local nominal strain. The local nominal strain is defined as the strain experienced by the median section of the hourglass sample during quasi-static deformation and was measured using an optical extensometer DIC. In order to measure the hysteresis loss, at first, the Mullins effect was eliminated by exposing the sample to 10 cycles, whereas the hysteresis loss was measured during the 10th cycle. In particular, the hysteresis loss was calculated by integrating the force versus displacement [19]. The utilized optical extensometer was based on stereo 3D digital image correlation (3D-DIC), which enables the strain components at the surface of the hourglass sample to be obtained. To perform 3D-DIC measurements, the sample was initially sprayed with white paint to create a stochastic speckle pattern. Images of the samples were recorded upon deformation with the help of a pair of 6-megapixel Schneider cameras. Post-processing was performed using Aramis GOM Correlate (Version 2018, Braunschweig, Germany) software. The true strain was obtained at the surface of the median section of the sample along in the radial and longitudinal directions. The logarithmic volume strain, which is defined as the logarithmic ratio between the current volume and the original volume of the sample at the median section, was computed using the equation [26]:

(3) $$\ln\left(\frac{\Delta V}{V_{0}}\right)=\ln(\lambda_{L})+2\ln(\lambda_{R})$$

where Δ*V* and *V*_0_ are the difference in the volume at a given time and initial volume, respectively, and *λ_L_* and *λ_R_* are the stretch ratio along the longitudinal and radial directions, respectively.

### 2.5. Dynamic Mechanical Analysis

The viscoelastic properties of the samples were measured using DMA in shear mode in the linear viscoelastic regime. Isothermal frequency sweeps at various temperatures covering both the glass transition and the rubber plateau were considered to construct master curves with respect to the time–temperature superposition principle [41]. The storage modulus (G’) and loss modulus (G’’) were recorded, and the loss tangent (tan δ) was calculated. The loss tangent, which is defined as the ratio of the loss modulus (G’’) to the storage modulus (G’), describes the amount of energy converted into heat (viscous part) to that recovered during the cyclic strain (elastic part).

The Payne effect measurements shown in this work were carried out using DMA in tension mode. These tests were performed at a constant frequency of 10 Hz with the dynamic strain sweep ranging from 0.01% to 10% at 21 °C.

### 2.6. Fatigue Testing and End of Life Criteria

Fatigue behavior was studied from hourglass-shaped samples. Displacement-controlled fatigue tests were performed applying a maximum local strain of 100%, 130%, 150%, or 200% and unloading the sample to a minimum local strain of zero. The tests were conducted using a servohydraulic fatigue testing system (Instron 8872, Norwood, MA, USA) equipped with a load cell of 5 kN. The sinusoidal displacement at the frequency of 2 Hz was used and carried out at a room temperature of 21 ± 2 °C. The end of the fatigue life (*N*_i_) of samples was determined as the number of fatigue cycles, at which the derivative of the load as a function of the number of cycles is not constant anymore. At the end, the Wöhler curves were generated [2,42]. The interrupted fatigue tests were performed for several percentages of *N*_i_. Only thereafter these fatigued samples were utilized for microscopic characterization.

### 2.7. Scanning Electron Microscopy

An SEM Quanta FEG 200 (FEI, Eindhoven, The Netherlands) was used to study the morphology of the cracks initiated on the sample surface. This SEM was operated in low vacuum mode, which allowed the analysis of samples without the need for any conductive coating. Interrupted fatigue samples were used for SEM observation. Before observing the sample by SEM, it was mounted onto a miniature tensile machine (Kammrath & Weiss, Schwerte, Germany), which was placed inside the SEM chamber. For facilitating the observation of the initiated cracks, samples were stretched with a displacement of 2 mm. Energy-dispersive X-ray spectroscopy (EDX Genesis XM 4i, EDAX Inc., Mahwah, NJ, USA) was used to verify the elemental nature of the crack initiation sites. Crack precursor size was obtained using SEM observations and was defined as the length of all the individual cracks detected on the sample surface.

### 2.8. Micro-Computed X-ray Tomography

A laboratory micro-computed X-ray tomograph (µCT) EasyTom 160 manufactured by RX Solutions (Chavanod, France) was employed for 3D imaging of the SRB composites (rectangular cuboid samples of dimension 2 mm × 2 mm × 5 mm and hourglass samples).

The hourglass samples were observed prior to, as well as after, mechanical testing, whereas the cuboid samples were only investigated prior to any mechanical testing. In the second case, the hourglass-shaped samples were stretched in the µCT with an applied displacement of 2 mm using a Deben CT5000 (Suffolk, UK) miniature mechanical testing device to reopen the cracks. To fully image the median section of the hourglass specimen, the source-to-object distance (SOD), as well as the source-to-detector distance (SDD), were adjusted in such a way as to obtain a voxel size of almost 9 µm. The acquisition parameters are summarized in Table 2. Following the 3D volume reconstruction with the Xact64 software (Version 2021, RX Solutions, Chavanod, France), a 3D image analysis of µCT projections was performed using the Avizo software (ThermoFisher, Waltham, MA, USA). Following subvolume extraction, a median filter was used to denoise the data. Next, following a segmentation step, only agglomerates and cracks with a size larger than 2.5 times the voxel size were considered for the analysis, given the error in determining the shape characteristics of the agglomerates and cracks [24]. The volume of the initiated cracks was calculated with respect to several stages of *N*_i_. A region of interest of 2 mm in height in the median section (highlighted in Figure 1) of the hourglass sample was considered for the volume fraction measurement.

## 3. Results and Discussions

### 3.1. Initial Morphology Characterization

**Figure 2:**
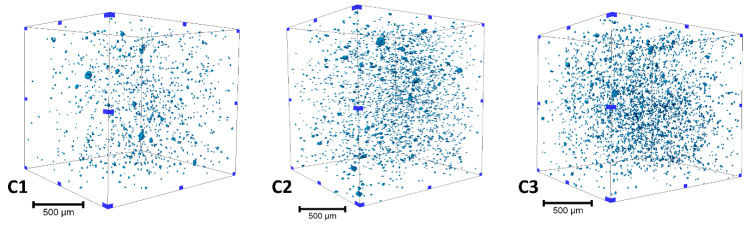
Volume rendering of the agglomerates in (**C1**–**C3**) based on µCT imaging and image analysis.

The morphological parameters of the agglomerates present in the different compounds were observed before performing fatigue testing by means of µCT. To this end, the µCT experiments were carried out on cuboid samples. The SOD and SDD parameters were adjusted in such a way that the sample was fully in the field of view and that the field of view of air volume surrounding the sample was reduced to a minimum. The statistical observation was carried out on a sample of volume 3.84 mm^3^ in which agglomerates of an equivalent diameter larger than 10.24 µm were considered. The dissimilarity in agglomerate dispersion is qualitatively documented by Figure 2, in which more agglomerates are present on the highest SSA compound (C3). A statistical analysis was carried out considering different morphological parameters, and the results are presented in Table 3. The median equivalent diameter of the agglomerates shows that the agglomerates were in a similar size range whatever the silica SSA. However, the volume fraction and density of the agglomerates increased with increasing silica SSA. This suggests that silica with a larger SSA has a greater tendency to reduce the silica dispersion state in the SBR matrix. Having a high SSA reduces the interaggregate distance, developing more interaction with their neighboring aggregates, and, therefore, it is more difficult for silica to be finely dispersed [43]. Additionally, undispersed agglomerates can be considered potential crack initiation sites, which would lead to a shorter product lifetime.

### 3.2. Crack Precursor Measurement Based on Tearing Energy

The stress–strain results based on quasi-static tests performed on dumbbell samples, as well as the force-displacement from the nicked angle tear tests results, are presented in Table 4. The sample’s crack precursors size was calculated by substituting stress at break, strain at break, and the critical tearing energy in Equation (2). From Table 4, the strain at break of the samples decreased with increasing silica SSA. The significant decrease in the strain value of the sample can be attributed to the increased interaction between the silica and SBR matrix when increasing SSA results in an increase in the stiffness of the material, as more stress is transferred to the reinforcing fillers. The crack precursor sizes of the three compounds are presented in Table 4. Values between 365 µm in the case of C1 and 501 µm in the case of C2 were obtained, which may indicate that C2 has the shortest and C1 has the longest fatigue life (large cracks promoting stress concentration) [31]. Fatigue testing will enable verification of this finding. However, this method is known to overestimate the real size of the crack precursors, as recently reported [31]. SEM imaging of cracks will enable us to confirm this point.

### 3.3. Viscoelastic Properties and Hysteresis

**Figure 3:**
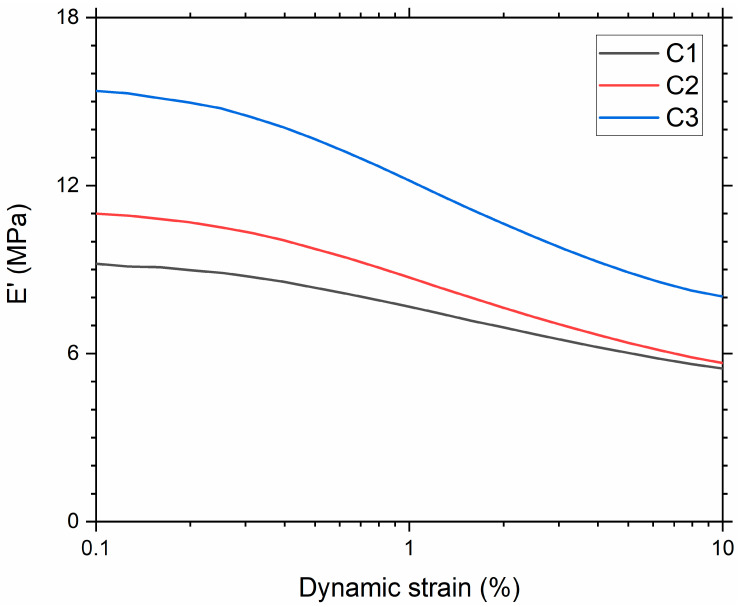
Storage modulus as a function of dynamic strain amplitude.

Figure 3 shows the dependence of the material’s storage modulus with respect to the dynamic strain. A typical Payne effect was noted for all the compounds, i.e., the storage modulus at the lowest strain amplitude decreased with an increase in strain. The magnitude of the Payne effect (∆E’) [44] is attributed to the degree of filler–filler breakdown (Table 5). The compound with the highest silica SSA showed the highest decrease in modulus and, therefore, the largest Payne effect. The largest Payne effect can be attributed to the increase in the filler network. In C3, the particle diameter and interaggregate distance decreased due to the increase in SSA. This facilitated the formation of a filler network. These filler networks are produced when the primary fused particles (aggregates) can agglomerate to create the filler network. The extent of the filler network is more noticeable with an increase in filler SSA [45], as confirmed by µCT imaging (Figure 2, Table 3).

**Figure 4:**
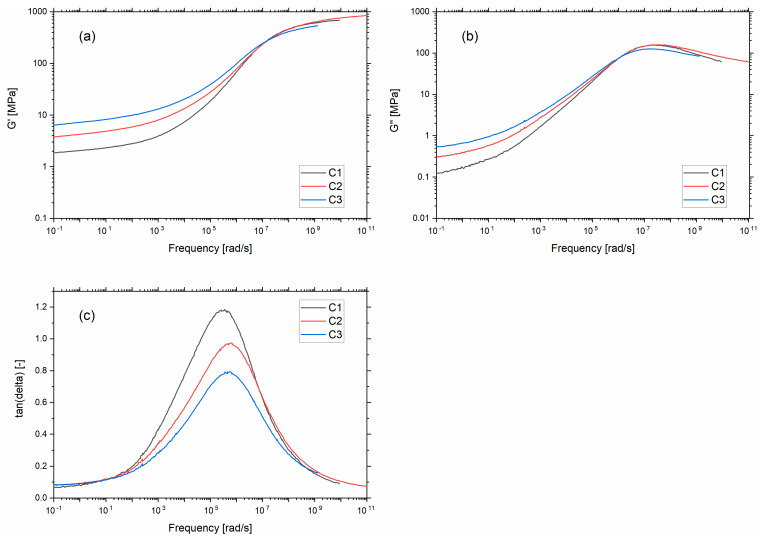
(**a**) Storage modulus (**b**) loss modulus and (**c**) tan δ versus angular frequency at 21 °C.

Following the Payne effect results, it is evident that the silica SSA has been perceived as a critical parameter in the material’s stiffness. Figure 4a,b shows the storage modulus (G’) and loss modulus (G”) master curves as a function of frequency at a reference temperature of 21 °C corresponding to the fatigue testing temperature. During these measurements, the percentage of dynamic strain used to stretch the samples was in the linear viscoelastic regime. In the rubbery regime (low-frequency range), an increase in G’ and G” is observed with increasing SSA. This can be explained by the formation of a rigid 3D filler network consisting of percolating paths of aggregates and agglomerates. µCT results showed that the increase in SSA promotes more interaction between fillers, meaning a stronger filler network. The increase in filler–filler interactions will induce an increase in G’ and G” due to the intrinsic higher stiffness of the filler (increase in G’) and by the amount of energy needed to alter the filler network (G”).

Figure 4c presents the frequency dependence of tan δ at 21 °C for the three compounds. Considering the transition regime, a decrease in the height of tan δ is observed as the silica SSA increases. Given that the α-relaxation in the transition zone is caused by cooperative motions of polymer chains, the decrease in peak height with increasing SSA can be attributed to the increase in local interactions between the polymer chains and the filler interface. These local interactions decrease the number of polymer chains participating in the molecular motions responsible for the α-relaxation process.

**Figure 5:**
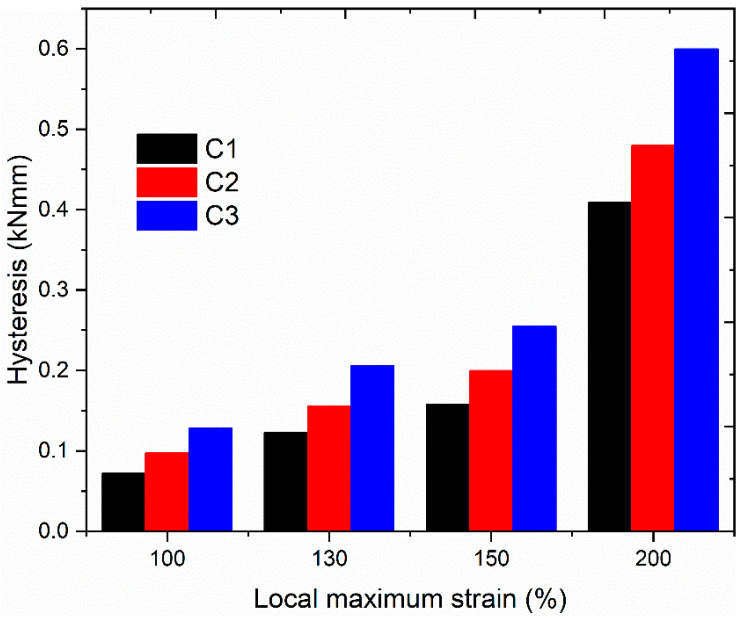
Measured hysteresis loss from compounds, including different silica fillers tested at different strain levels.

Samples with hourglass geometry (see Figure 1) were used for the hysteresis loss measurement. Hysteresis losses of compounds with different SSAs are shown in Figure 5. Here again, the hysteresis losses increase with increasing local maximum strain and silica SSA. For a constant filler content, silica fillers with larger SSA, i.e., smaller particle size, facilitate the formation of a secondary filler–filler structure, i.e., agglomerates [18]. Therefore, the disaggregation of agglomerates during deformation increases with an increase in SSA [18]. As a result, hysteresis loss increases and contributes to a larger heat generation in the compound with increasing SSA.

### 3.4. Fatigue Life

**Figure 6:**
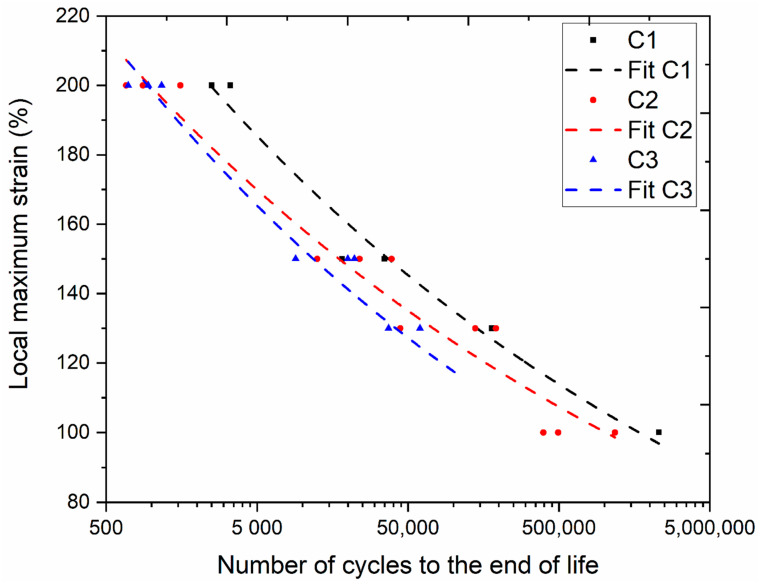
Generated Wöhler curves for compounds C1, C2, and C3.

For comparing compound fatigue properties, Wöhler curves were generated by correlating the number of cycles until the end of life with different local strain levels (see Figure 6). A power-law function (y = a·x^b^) was used to fit the data. The local maximum strain, as presented in Figure 6, was measured on the surface of the median section of the sample using 3D-DIC. As expected, the fatigue life of the compound increased with reducing the imposed local surface strain. Regardless of the local maximum strain to which the sample was subjected, fatigue life decreased from C1 to C3, indicating that the estimated crack precursor sizes by tearing testing (Table 4) are not reflecting this fatigue life order (C2 was estimated to have the largest crack precursor size and, hence, the shortest fatigue life). Whereas previous studies have shown that the fatigue life of filled compounds depends on the test conditions, load ratios [42], and additives [8], this study implies that the compound’s fatigue life also depends on the SSA of the silica filler. The improvement in the fatigue end of life could be due to the reduction in stiffness of the material, as stiffness decreased with the decrease in the SSA; thus, requiring less energy for the cyclic deformation. Generally, an increased energy input results in a decreased fatigue end of life [10]. Additionally, from Table 3, the volume fraction of agglomerate in C1 is lower in comparison to C3. Therefore, the shortest distance between the two agglomerates will be more significant compared with C3. It is reported that a decrease in the distance between two agglomerates drastically decreases the strain for the cavitation [33]. Thus, there could be a delay in losing the structural stability of the compound, hence improving fatigue end of life.

### 3.5. Mechanisms of Crack Initiation

**Figure 7:**
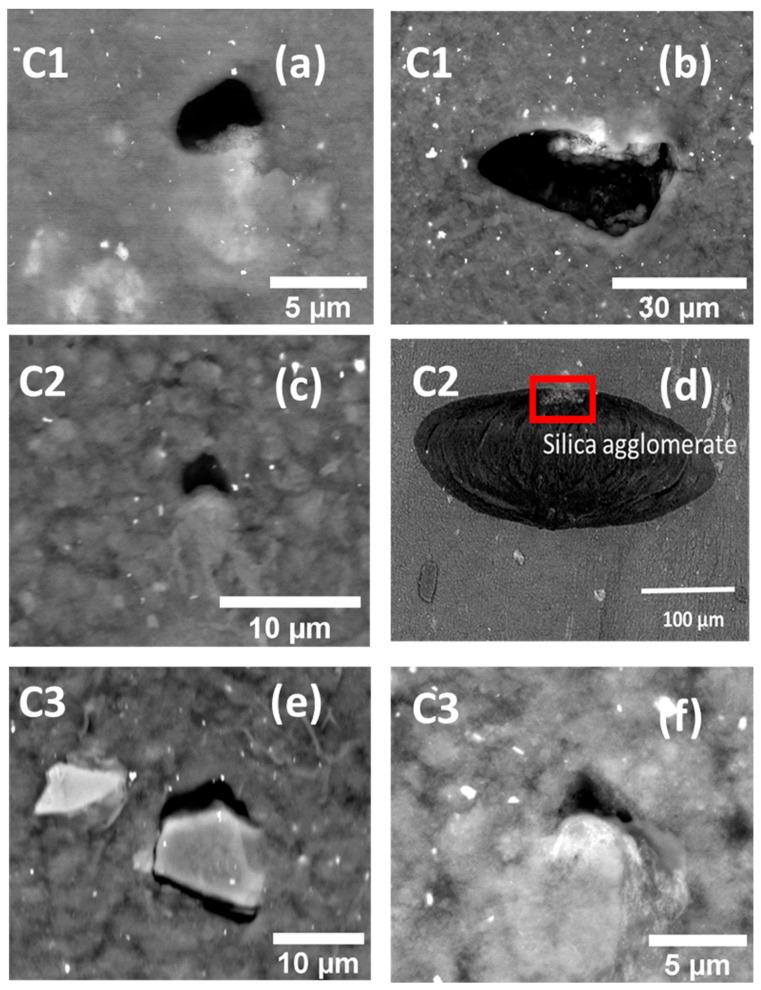
SEM backscattered electron imaging of interrupted fatigue sample surface showing debonding at one pole and at both poles for various compounds at stage 1 (**a**,**c**,**e**,**f**), stage 2 (**b**) and stage 3 (**d**) respectively (samples subjected to 200% of local strain and 40% of *N*_i_, loading direction is vertical).

SEM analyses were performed on the surface of the interrupted fatigue samples to understand the mechanisms of crack initiation. While performing the SEM measurements, the sample was stretched by 2 mm using a miniature tensile machine for reopening the cracks induced by the fatigue testing. Figure 7 shows debonded silica agglomerates from the SBR matrix. The detachment zone is at the poles of the agglomerates, and the crack propagates perpendicular to the loading direction. In addition, there were significant morphological similarities observed between the agglomerates induced debonding mechanisms. Thus, from the 2D images, it appears that in some observed debonding sites (e.g., Figure 7e), the silica agglomerates have a sharper edge at the debonded pole, at least in two dimensions. It is generally known that a sharper edge raises the stress concentration and facilitates crack initiation at the interface. Therefore, it can be rationalized that the crack initiation of silica-filled SBR composites under dynamic loading originates from filler–matrix debonding. From the SEM observations, three stages of crack evolution at the vicinity of silica agglomerates were identified. These stages were similar for the three compounds of the study. In stage 1 (Figure 7a), the initiation of crack was noted due to matrix–agglomerate debonding occurring at the agglomerate poles. Figure 7b represents stage 2 characterized by the sidewise opening of the initiated crack, whereas stage 3 (Figure 7d) corresponds to the fast growth of the crack on the surface and in the bulk perpendicular to the loading direction, expected to form at a later stage a macro-crack conducting to the sample fracture.

**Figure 8:**
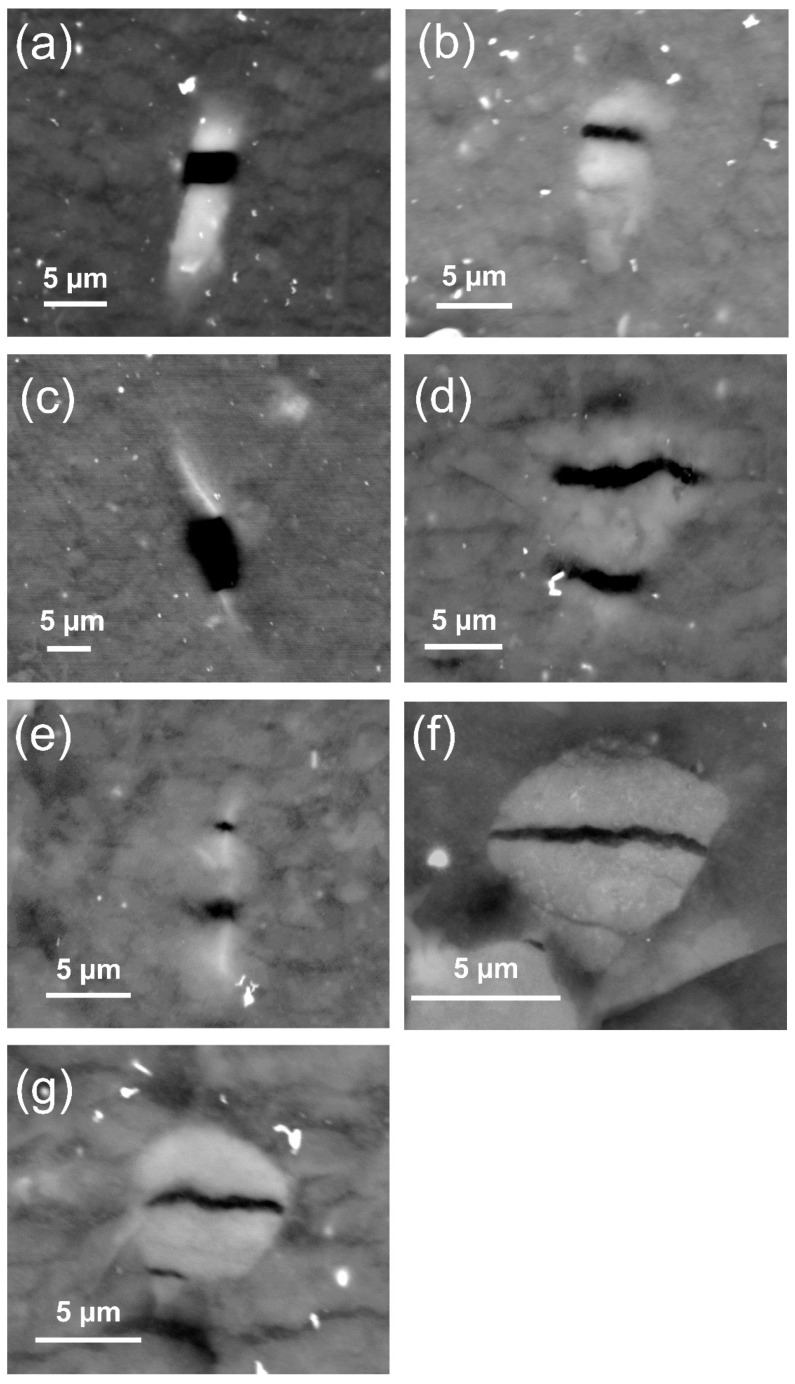
SEM backscattered electron imaging of interrupted fatigue sample surface showing internal silica agglomerate fracture at one visible (**a**–**c**) or multiple (**d**–**g**) locations (200% of local strain and 40% of *N*_i_, loading direction is vertical).

Apart from the debonding mechanism, another specific initiation mechanism was identified for the samples subjected to interrupted fatigue tests. Indeed, some silica agglomerates fractured into two or more fragments, as depicted in Figure 8. This could be due to the fracture of agglomerates during the sample preparation process or to pre-existing internal flaws [46]. Defining the aspect ratio of the agglomerate as the ratio of their vertical length i.e., the length parallel to the loading direction, to the agglomerate’s horizontal length [47], it can be seen from Figure 8 that the apparent correlation between the shape, orientation, and alignment of the agglomerates was causing the crack initiation. We observed from the SEM micrographs that the silica agglomerates of aspect ratio greater than one (i.e., the longest length is oriented along the loading direction) were mostly fractured into halves. This suggests that a significant particle orientation effect is present. Thus, particles with a large aspect ratio (>1) are more susceptible to internal agglomerate fracture than axisymmetric particles (if aspect ratio equals 1).

**Figure 9:**
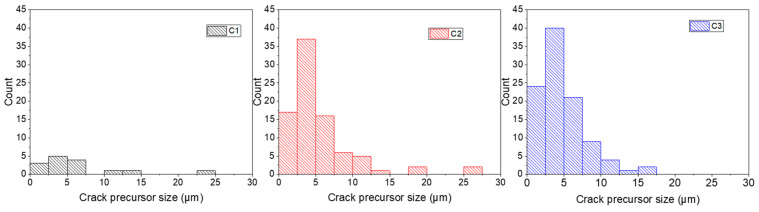
Distribution of crack precursor size observed on the different compounds subjected to 200% of local strain and 40% of *N_i_*.

The crack precursor size was determined by quantitative analysis of SEM images (Figure 9). For the calculations, crack precursors resulting from both the matrix–agglomerate debonding and the internal fracture of the agglomerate were considered. The result shows that the majority of the crack precursors had a size below 10 µm for C1 to C3. It was also noted in the three compounds that the cracks initiated from silica agglomerates having a size between 20 µm and 50 µm tend to propagate very fast and, hence, were considered critical cracks. Moreover, the calculated crack precursor sizes obtained by SEM imaging were different from those determined from the critical tearing energy method (Table 4). In particular, the values obtained by quantitative microscopy were much smaller than the ones obtained from the mechanical testing method. Considering that the direct observation of the crack is much more accurate than the second method, we conclude that the critical tearing energy method markedly overestimates the crack precursor size, confirming a previous study [31]. This can be explained by the fact that the used macroscopic mechanical testing method is not sensitive enough to crack initiation occurring at the microscopic scale. 

### 3.6. Volume of Cracks Based on µCT and DIC

**Figure 10:**
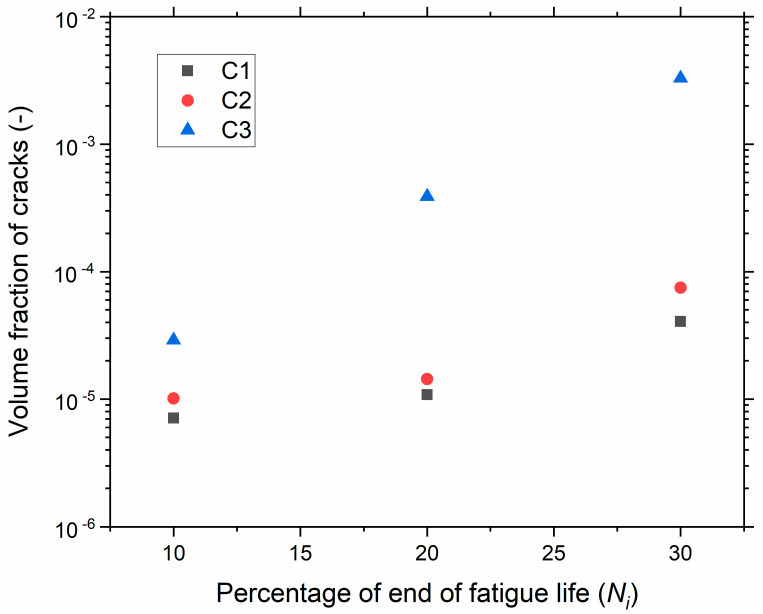
Volume fraction of cracks versus percentage of end of life using µCT.

The crack volume fraction, i.e., the ratio of the volume of the crack to the total volume of the sample, was assessed by performing µCT on the hourglass samples for the strain level 150% for different compounds. The volume of cracks is presented in Figure 10 for different levels of fatigue end life (*N*_i_). Most of the cracks initiated in the surface and sub-surface regions of the sample’s median section. Here again, it was observed that the cracks mainly initiated due to debonding and internal fracture of the silica agglomerates. C1 and C2 had a higher resistance to crack initiation; the volume fraction of cracks in C3 was around three times higher than for C1 and C2 and continued to rise at a faster rate with increasing *N*_i_. The volume of the cracks increased with increasing numbers of cycles to rupture, and the cracks initiated at later stages of fatigue life. Further, cracks grew and coalesced, leading to fracture.

**Figure 11:**
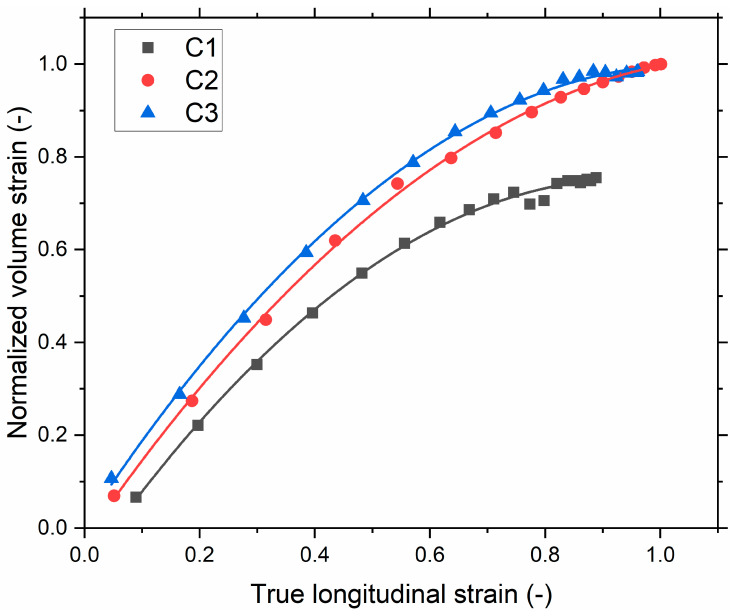
Normalized volume strain versus true longitudinal strain by DIC (computed for the samples being subjected to 200% of local maximum strain at the 10th loading cycle) and a polynomial fit is used.

A quantitative comparison was made with the DIC results. The normalized volume strain, obtained by dividing the measured volume strain of each compound by the global maximum volume strain, of the different compounds determined using 3D-DIC measurement is shown in Figure 11. The choice of normalizing the volume strain comes from the intrinsic gradient of deformation in the hourglass geometry, being the maximum strain at the surface and the minimum at the center. Therefore, a precise determination of the deformation is not ensured with the current method, inducing experimental bias in calculating the volume strain. By normalizing, we propose to discuss the overall trends of the volume increase measured at the surface of the hourglass specimens. Figure 11 shows the stretch dependencies of the normalized volume strain for all three investigated compounds. Upon stretching, a noticeable increase in the normalized volume strain is observed. The normalized volume strain of the compounds depends on the SSA of the filler: the higher the SSA, the higher the volume strain. The µCT and 3D-DIC results show a similar trend for the various compounds. Thus, the evaluation of the volume change can be a suitable approach to understand the incompressibility. The rise in the macroscopic volume can be correlated to the initiation and propagation of cracks. Therefore, this method suggests that the µCT and 3D-DIC results are qualitatively comparable [24,25]. Thus, it demonstrates the relevancy of two methods on the durability-based ranking of composites during deformation.

## 4. Conclusions

This study focused on understanding the influence of silica SSA on the fatigue and viscoelastic behavior of SBR/silica composite. From the DMA analysis and hysteresis loss testing, the stiffness of the compounds and the hysteresis loss increase with the SSA, which is attributed to the increase in the reinforcement effect of high SSA silica and the increase in the degree of breakdown of filler network upon deformation. By µCT, the volume fraction of the agglomerates was investigated. It was observed that the detected volume fraction of the agglomerates increases with the SSA, which is related to the strong filler network. These filler networks, i.e., agglomerates, are being considered crack initiation sites. SEM observations on interrupted fatigue samples revealed two types of crack initiation mechanisms initiated from the silica agglomerates: (a) matrix silica interfacial debonding at one or both poles of the fillers and (b) internal silica. The detected initial cracks were oriented perpendicular to the loading direction. These mechanisms were observed in the case of all the compounds irrespective of the SSA. Moreover, it was observed that the cracks initiated even before 10% of fatigue end life. Based on the SEM observations on the sample surface, we found that most of the crack precursors had a size below 10 µm, whereas the tearing testing method provided much higher values of crack precursor size. The influence of SSA on fatigue end life was characterized by generating the Wöhler curve for different strain levels. The latter indicates that the fatigue end life decreases with an increase in the SSA. This can be corroborated by the increase in the volume fraction of the filler network. Hence, an increase in stiffness could be related to a stronger filler–filler interaction. Some calculations of the volume strain with DIC measurements revealed the same trend as the volume strain determined by µCT experiments. Indeed, for the two volume strain calculation methods, the volume strain increased with SSA. Microscale structural characterization methods indicate that the macroscopic rise in volume strain observed by DIC coincides with the formation and growth of cracks. The decrease in fatigue life with increasing SSA was accompanied by the rise in hysteresis loss and stiffness. It is concluded that the SSA plays a predominant role in the compound’s fatigue life and viscoelastic properties and, therefore, has a profound influence on the applications for which it is developed. Thus, it may be beneficial to use a silica-filled rubber material with a large SSA in an elastomeric product where the rigidity is essential and where the material is not exposed to a high number of fatigue cycles, whereas a silica-filled rubber material with a low SSA would be used in an elastomeric product where the rigidity is less important, but the fatigue properties are essential.

## Figures and Tables

**Table 1:** Compound formulations.

		Compound 1	Compound 2	Compound 3
N_2_ Silica specific surface area, BET (m^2^/g)		125	165	200
	SBR SE SLR 4602 *	100	100	100
	Antioxidant	2.5	2.5	2.5
	Oil Vivatec 500	25	25	25
	Activator Stearic Acid	3	3	3
	Silica Hi-Sil 315 **	90	-	-
Ingredients (phr)	Silica Zeosil 1165 MP ***	-	90	-
	Silica Zeosil Premium 200 MP ***	-	-	90
	Silane Si266	6	7.2	9
	ZnO	2.5	2.5	2.5
	Sulfur	1.4	1.4	1.4
	Accelerator	1.8	2.3	2.88
	Accelerator	2.5	3.2	4
Total phr		234.7	237.1	240.28

* Trinseo, Berwyn, PA, USA; ** PPG, Monroeville, PA, USA; *** Solvay, Paulínea, SP, Brazil.

**Table 2:** µCT parameters.

Acquisition Parameter	Hourglass Sample	Cuboid Sample
SOD (mm)	35	7.75
SDD (mm)	497	329
Voxel size (µm)	9	3
X-ray source voltage (kV)	45	40
X-ray source current (µA)	165	160
Frame rate (frame/s)	1	0.75
Number of averaged frames	5	3
Angular range (°)	360	360
Angular step (°)	0.25	0.25

**Table 3:** 3D image analysis of the agglomerates in C1 to C3.

	C1	C2	C3
Median equivalent diameter (µm)	12.42	12.05	12.10
Volume fraction (%)	0.12	0.24	0.27
Density of agglomerates per mm^3^	307	746	896

**Table 4:** Mechanical properties and crack precursor size for the tested samples.

	Stress at Break (MPa)	Strain at Break (%)	Tear Strength (N/mm)	Crack Precursor Size (µm)
C1	18.2 ± 0.1	277.0 ± 5.7	30.0 ± 0.5	365.0 ± 0.7
C2	15.6 ± 0.4	273.0 ± 2.1	35.3 ± 0.2	501.0 ± 16.9
C3	19.0 ± 0.2	254.0 ± 0.7	29.9 ± 0.7	370.0 ± 4.9

**Table 5:** Magnitude of Payne effect.

	E’(0.1)	E’(10)	∆E’ [E’(0.1)–E’(10)]
C1	9.2	5.5	3.7
C2	10.9	5.7	5.2
C3	15.3	8.0	7.3

## Data Availability

Not applicable.

## References

[1] Mars W.V., Fatemi A. (2003). Fatigue crack nucleation and growth in filled natural rubber. Fatigue Fract. Eng. Mater. Struct..

[2] Huneau B., Masquelier I., Marco Y., Le Saux V., Noizet S., Schiel C., Charrier P. (2016). Fatigue crack initiation in a carbon black–filled natural rubber. Rubber Chem. Technol..

[3] Studebaker M.L. (1957). The Chemistry of Carbon Black and Reinforcement. Rubber Chem. Technol..

[4] Stockelhuber K., Svistkov A., Pelevin A., Heinrich G. (2011). Impact of filler surface modification on large scale mechanics of styrene butadiene/silica rubber composites. Macromolecules.

[5] Robertson C.G., Hardman N.J. (2021). Nature of Carbon Black Reinforcement of Rubber: Perspective on the Original Polymer Nanocomposite. Polymers.

[6] Yang R., Song Y., Zheng Q. (2017). Payne effect of silica-filled styrene-butadiene rubber. Polymer.

[7] Payne A., Whittaker R. (1971). Low strain dynamic properties of filled rubbers. Rubber Chem. Technol..

[8] Gauthier C., Reynaud E., Vassoille R., Ladouce-Stelandre L. (2004). Analysis of the non-linear viscoelastic behaviour of silica filled styrene butadiene rubber. Polymer.

[9] Mars W.V., Fatemi A. (2002). A literature survey on fatigue analysis approaches for rubber. Int. J. Fatigue.

[10] Zhao J., Ghebremeskel G.N. (2001). A Review of Some of the Factors Affecting Fracture and Fatigue in SBR and BR Vulcanizates. Rubber Chem. Technol..

[11] Zine A., Benseddiq N., Naït Abdelaziz M. (2011). Rubber fatigue life under multiaxial loading: Numerical and experimental investigations. Int. J. Fatigue.

[12] Ruellan B., Le Cam J.B., Jeanneau I., Canévet F., Mortier F., Robin E. (2019). Fatigue of natural rubber under different temperatures. Int. J. Fatigue.

[13] Ostoja-Kuczynski E., Charrier P., Verron E., Marckmann G., Gornet L., Chagnon G. Crack initiation in filled natural rubber: Experimental database and macroscopic observations. Proceedings of the Third European Conference on Constitutive Models for Rubber.

[14] Tee Y.L., Loo M.S., Andriyana A. (2018). Recent advances on fatigue of rubber after the literature survey by Mars and Fatemi in 2002 and 2004. Int. J. Fatigue.

[15] Mullins L. (1948). Effect of Stretching on the Properties of Rubber. Rubber Chem. Technol..

[16] Diani J., Fayolle B., Gilormini P. (2009). A review on the Mullins effect. Eur. Polym. J..

[17] Weng G., Yao H., Chang A., Fu K., Liu Y., Chen Z. (2014). Crack growth mechanism of natural rubber under fatigue loading studied by a real-time crack tip morphology monitoring method. RSC Adv..

[18] Kar K.K., Bhowmick A.K. (1997). Hysteresis loss in filled rubber vulcanizates and its relationship with heat generation. J. Appl. Polym. Sci..

[19] Kim J.H., Jeong H.Y. (2005). A study on the material properties and fatigue life of natural rubber with different carbon blacks. Int. J. Fatigue.

[20] Thaptong P., Sae-oui P., Sirisinha C. (2017). Influences of styrene butadiene rubber and silica types on performance of passenger car radial tire tread. Rubber Chem. Technol..

[21] Robertson C.G., Lin C.J., Bogoslovov R.B., Rackaitis M., Sadhukhan P., Quinn J.D., Roland C.M. (2011). Flocculation, reinforcement, and glass transition effects in silica-filled styrene-butadiene rubber. Rubber Chem. Technol..

[22] Choi S.-S. (2001). Improvement of properties of silica-filled styrene–butadiene rubber compounds using acrylonitrile–butadiene rubber. J. Appl. Polym. Sci..

[23] Ramier J., Gauthier C., Chazeau L., Stelandre L., Guy L. (2007). Payne effect in silica-filled styrene–butadiene rubber: Influence of surface treatment. J. Polym. Sci. Part B Polym. Phys..

[24] Federico C.E., Padmanathan H.R., Kotecky O., Rommel R., Rauchs G., Fleming Y., Addiego F., Westermann S., Heinrich G., Kipscholl R., Stoček R. (2020). Cavitation Micro-mechanisms in Silica-Filled Styrene-Butadiene Rubber upon Fatigue and Cyclic Tensile Testing. Fatigue Crack Growth in Rubber Materials.

[25] Federico C.E., Rauchs G., Kotecky O., Westermann S., Addiego F. (2020). Cavitation in thermoplastic-reinforced rubber composites upon cyclic testing: Multiscale characterization and modelling. Polymer.

[26] Andersen M., Hopperstad O.S., Clausen A.H. (2019). Volumetric strain measurement of polymeric materials subjected to uniaxial tension. Strain.

[27] Starkova O., Aniskevich A. (2010). Poisson’s ratio and the incompressibility relation for various strain measures with the example of a silica-filled SBR rubber in uniaxial tension tests. Polym. Test..

[28] Robertson C.G., Tunnicliffe L.B., Maciag L., Bauman M.A., Miller K., Herd C.R., Mars W.V. (2020). Characterizing Distributions of Tensile Strength and Crack Precursor Size to Evaluate Filler Dispersion Effects and Reliability of Rubber. Polymer.

[29] Li F., Liu J., Mars W.V., Chan T.W., Lu Y., Yang H., Zhang L. (2015). Crack precursor size for natural rubber inferred from relaxing and non-relaxing fatigue experiments. Int. J. Fatigue.

[30] Ludwig M., Alshuth T., El Yaagoubi M., Juhre D., Marvalova B., Petrikova I. (2015). Lifetime prediction of elastomers based on statistical occurrence of material defects. Constitutive Models for Rubbers IX.

[31] Guo H., Li F., Wen S., Yang H., Zhang L. (2019). Characterization and Quantitative Analysis of Crack Precursor Size for Rubber Composites. Materials.

[32] Chakraborty S.K., De S.K. (1982). Silica- and Clay-Reinforced Carboxylated Nitrile Rubber Vulcanized by a Mixed Crosslinking System. Rubber Chem. Technol..

[33] Glanowski T., Huneau B., Marco Y., Le Saux V., Champy C., Charrier P. (2018). Fatigue initiation mechanisms in elastomers: A microtomography-based analysis. MATEC Web Conf..

[34] Marco Y., Huneau B., Masquelier I., Le Saux V., Charrier P. (2017). Prediction of fatigue properties of natural rubber based on the descriptions of the cracks population and of the dissipated energy. Polym. Test..

[35] Marco Y., Masquelier I., Le Saux V., Calloch S., Huneau B., Charrier P. Contributions of IR thermography and X-ray tomography to the fatigue characterization of elastomeric materials. Proceedings of the ECCMR VIII.

[36] Weng G., Chang A., Fu K., Kang J., Ding Y., Chen Z. (2016). Crack growth mechanism of styrene-butadiene rubber filled with silica nanoparticles studied by small angle X-ray scattering. RSC Adv..

[37] Balutch T., Huneau B., Marco Y., Charrier P., Champy C. (2018). Fatigue behaviour of an industrial synthetic rubber. MATEC Web Conf..

[38] Marco Y., Le Saux V., Calloch S., Charrier P. (2010). X-ray computed μ-tomography: A tool for the characterization of fatigue defect population in a polychloroprene rubber. Procedia Eng..

[39] Guy L., Daudey S., Cochet P., Bomal Y. (2009). New insights in the dynamic properties of precipitated silica filled rubber using a new high surface silica. Kautsch. Gummi Kunstst..

[40] Kaewsakul W., Sahakaro K., Dierkes W.K., Noordermeer J.W. (2012). Optimization of mixing conditions for silica-reinforced natural rubber tire tread compounds. Rubber Chem. Technol..

[41] Leblanc J.L., Cartault M. (2001). Advanced torsional dynamic methods to study the morphology of uncured filled rubber compounds. J. Appl. Polym. Sci..

[42] Belkhiria S., Seddik R., Atig A., Ben Sghaier R., Hamdi A., Fathallah R. (2018). Fatigue reliability prediction of rubber parts based on Wöhler diagrams. Int. J. Adv. Manuf. Technol..

[43] Donnet J.-B., Custodero E., Mark J.E., Erman B., Eirich F.R. (2005). 8-Reinforcement of Elastomers by Particulate Fillers. Science and Technology of Rubber.

[44] Luginsland H.-D., Frohlich J., Wehmeier A. (2002). Influence of Different Silanes on the Reinforcement of Silica-Filled Rubber Compounds. Rubber Chem. Technol..

[45] Fröhlich J., Niedermeier W., Luginsland H.D. (2005). The effect of filler–filler and filler–elastomer interaction on rubber reinforcement. Compos. Part A Appl. Sci. Manuf..

[46] Koh S.-W., Kim J.-K., Mai Y.-W. (1993). Fracture toughness and failure mechanisms in silica-filled epoxy resin composites: Effects of temperature and loading rate. Polymer.

[47] Hosseinpour K., Ghasemi A.R. (2021). Agglomeration and aspect ratio effects on the long-term creep of carbon nanotubes/fiber/polymer composite cylindrical shells. J. Sandw. Struct. Mater..

